# Supplementary material for: Impact of Water Regimes and Amendments on Inorganic Arsenic Exposure to Rice
Source: Int J Environ Res Public Health. 2021 Apr 27;18(9):4643. doi: 10.3390/ijerph18094643 (PMC8123884; doi:10.3390/ijerph18094643)
Supplement: Supplementary file 1 [file ijerph-18-04643-s001.zip › ijerph-1181724-supplementary.pdf]

Supplementary tables

Table S1. Initial physicochemical properties of experimental soil

Parameters	
Sand (%)	25.2
Silt (%)	34.9
Clay (%)	39.9
Texture	Clay loam
pH	7.59
Organic carbon (%)	0.81
Amorphous Fe (g kg^{-1})	1.92
Total As (mg kg^{-1})	11.62

Data represents mean of triplicate analysis, values varied by less than 5%

Table S2. Comparisons between certified and measured values of reference material.

SRM		Inorganic As	Total As
NIST 1568b	Measured value	0.087 ± 0.006	0.268 ± 0.011
	Certified value	0.092	0.291

Table S3. Recovery (%) of different arsenic species and total arsenic in different sample matrices.

Sample Type	Spike Level (mg/kg)	Arsenite	Arsenate	Total As
Grain (*n* = 6)	0.02	97.4 ± 3.4	91.6 ± 3.8	98 ± 5.0

Table S4. Available Si and amorphous Fe oxide concentration in soil subject to water regime and soil amendment

Water regime	Soil amendment	Available Si (mg/kg)	Amorphous Fe oxide (mg/kg)
Flooded	Control	340	1560
	Silicon + NPK	384	1482
	Iron + NPK	388	1944
	Silicon + FYM	392	1380
	Iron + FYM	372	2274
	Silicon + VC	404	1746
	Iron + VC	336	1980
Aerobic	Control	420	2076
	Silicon + NPK	488	2815
	Iron + NPK	452	3022

	Silicon + FYM	464	2753
	Iron + FYM	472	3298
	Silicon + VC	512	2539
	Iron + VC	464	3353
